# Supplementary material for: Toxicity of Silver Nanoparticles Supported by Surface-Modified Zirconium Dioxide with Dihydroquercetin
Source: Nanomaterials (Basel). 2022 Sep 14;12(18):3195. doi: 10.3390/nano12183195 (PMC9502449; doi:10.3390/nano12183195)
Supplement: Supplementary file 1 [file nanomaterials-12-03195-s001.zip › nanomaterials-1921721-supplementary.pdf]

nanomaterials

Article

Toxicity of Silver Nanoparticles Supported by Surface-Modified Zirconium Dioxide with Dihydroquercetin

Dušan Sredojević [1,2], Vesna Lazić [1,*], Andrea Pirković [3], Jovana Periša [1], Natalija Murafa [4], Biljana Spremo-Potparević [5], Lada Živković [5], Dijana Topalović [5], Aleksandra Zarubica [6], Milica Jovanović Krivokuća [3] and Jovan M. Nedeljković [1,*]

[1] Centre of Excellence for Photoconversion, Vinča Institute of Nuclear Sciences—National Institute of the Republic of Serbia, University of Belgrade, 11000 Belgrade, Serbia
[2] Department of Science, Texas A&M University at Qatar, Doha P.O. Box 23874, Qatar
[3] Department for Biology of Reproduction, INEP Institute for Application of Nuclear Energy, University of Belgrade, 11000 Belgrade, Serbia
[4] Institute of Inorganic Chemistry of the Czech Academy of Sciences, 250 68 Husinec-Řež, Czech Republic
[5] Department of Pathobiology, Faculty of Pharmacy, University of Belgrade, 11000 Belgrade, Serbia
[6] Department of Chemistry, Faculty of Science and Mathematics, University of Niš, Višegradska 33, 18000 Niš, Serbia
* Correspondence: vesna.lazic@vin.bg.ac.rs (V.L.); jovned@vin.bg.ac.rs (J.M.N.)

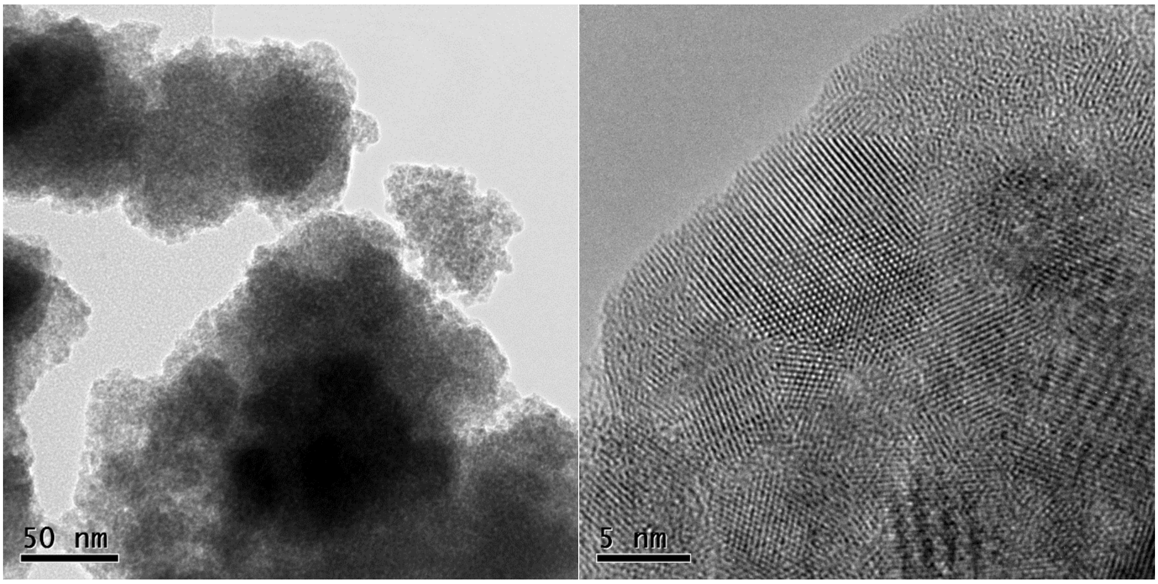

Figure S1. Low- and high-resolution TEM images of ZrO_2/DHQ.
